# A review of feed efficiency in swine: biology and application

**DOI:** 10.1186/s40104-015-0031-2

**Published:** 2015-08-06

**Authors:** John F. Patience, Mariana C. Rossoni-Serão, Néstor A. Gutiérrez

**Affiliations:** Department of Animal Science, Iowa State University, Ames, IA 50011-3150 USA

**Keywords:** Caloric efficiency, Energy, Feed efficiency, Residual feed intake, Swine

## Abstract

Feed efficiency represents the cumulative efficiency with which the pig utilizes dietary nutrients for maintenance, lean gain and lipid accretion. It is closely linked with energy metabolism, as the oxidation of carbon-containing components in the feed drive all metabolic processes. While much is known about nutrient utilization and tissue metabolism, blending these subjects into a discussion on feed efficiency has proven to be difficult. For example, while increasing dietary energy concentration will almost certainly increase feed efficiency, the correlation between dietary energy concentration and feed efficiency is surprisingly low. This is likely due to the plethora of non-dietary factors that impact feed efficiency, such as the environment and health as well as individual variation in maintenance requirements, body composition and body weight.

Nonetheless, a deeper understanding of feed efficiency is critical at many levels. To individual farms, it impacts profitability. To the pork industry, it represents its competitive position against other protein sources. To food economists, it means less demand on global feed resources. There are environmental and other societal implications as well.

Interestingly, feed efficiency is not always reported simply as a ratio of body weight gain to feed consumed. This review will explain why this arithmetic calculation, as simple as it initially seems, and as universally applied as it is in science and commerce, can often be misleading due to errors inherent in recording of both weight gain and feed intake.

This review discusses the importance of feed efficiency, the manner in which it can be measured and reported, its basis in biology and approaches to its improvement. It concludes with a summary of findings and recommendations for future efforts.

## Introduction

Feed represents between 60 and 70 % of the total cost of pork production in modern capital-intensive systems. Within feed, energy alone may represent 50 % or more of the total cost [1]. Carbon-containing compounds in the feed, including fat, carbohydrate and protein, release energy when oxidized. Such energy is required for such processes as the biosynthesis of proteins, bones and lipids, for biochemical processes associated with maintenance, for active ion transport and for mechanical work [2].

Energy is the critical dietary constituent that supports maintenance, as well as tissue accretion, and knowledge of energy metabolism and growth is essential to the understanding of feed efficiency [1]. In general terms, the immature pig will typically attempt to consume sufficient feed to meet its energy requirement for maintenance and growth. Yet, in many situations, feed intake is impaired by social, physiological, or environmental constraints. As a result, daily energy intake often falls short of that needed to support maximal gain. Consequently, feed and energy intake are highly complex subjects, and despite decades of research, gaps in our understanding persist [3].

While feed efficiency strongly influences financial returns [4], due to its close association with feed costs, actions taken to improve feed efficiency can inadvertently lead to financial losses rather than gains. This is due to the fact that single-minded actions taken to improve feed efficiency may affect other aspects of the enterprise, not the least of which is the cost of feed. As one example, genetic selection solely for improved feed efficiency may reduce growth rate [5]. Another example would be increasing dietary energy concentration; while this simple action will almost always increase feed efficiency, it could actually increase feed cost per pig and concurrently lower net income.

For such an important subject, feed efficiency is often misunderstood, and there is little universal agreement on the best approaches to achieving optimal feed efficiency. Part of this confusion arises from the unfortunate fact that the biological basis of feed efficiency is poorly understood [1]. There is also disagreement – and misunderstanding – on how to measure and express feed efficiency; for example, expressing feed efficiency on a live weight gain basis can result in misleading conclusions [6]. Finally, it needs to also be recognized that effectively measuring feed efficiency can be extremely difficult.

### Measuring feed efficiency

A common and simple definition of feed efficiency in the scientific literature is body weight gain per unit of feed consumed. Sometimes, feed efficiency is expressed on a dietary energy basis rather than feed intake. Although the concept is fairly simple, beneath it lays the potential for a multitude of practical errors. For example, feed consumed is rarely measured; feed disappearance is the actual measurement. Because of differences in feeder design and feeder adjustment, feed consumed and feed disappeared can differ by 10 % and sometimes by as much as 30 % [7]. It is therefore important to realize that feed disappearance does not necessarily accurately reflect feed intake by the pig and improving feed efficiency in a particular circumstance may be as simple as feeder adjustment or redesign.

The weight range in which feed conversion is measured is also of critical importance [4]. Animals of different weights have different requirements for maintenance; therefore, when comparing groups of pigs for feed efficiency, the variation due to differences in body weights at which various animals are evaluated must be taken into account. Equations that can be used to adjust for differences in initial or final bodyweight are available [4]. This has implications in both research and commercial application. Unfortunately, many experiments are conducted to a common time endpoint, rather than a common body weight endpoint, making comparison of feed efficiency across treatments very difficult, if not impossible.

Energetic differences in diets can also introduce unexpected, or even unseen, errors. Some of this error can be due to inaccurate estimates of dietary energy or due to the energy system being used for the measurement. Thus, differences in measured feed efficiency may be the consequence of unmeasured differences in dietary energy; this can become a particularly serious problem when diets widely varying in protein, lipid or fiber content are being compared. Furthermore, variation in the composition of gain (fat, lean, or bone) and in related maintenance requirements prevents this measure from being a precise estimate of energy conversion rate. Maximum lean growth potential, as well as fat deposition rates, vary as a function of feed intake among genotypes and sex [8]. Therefore, the most useful criteria to evaluate feed efficiency in meat producing animals should be the amount of edible product achieved with a given energy intake, rather than the fraction of energy in the feed which was converted to total body weight [9].

In studies of genetic variance and heritability, and especially in selection programs, traditional measures of feed efficiency treat animals with differing growth rates and differing mean body weights equally [9]. In an attempt to more effectively compare individual animals, Koch et al. adjusted feed consumed for rate of gain and for mean body weight; this new approach evolved in swine to measuring growth rate as well as body composition and became known as residual feed intake (RFI). Residual feed intake is calculated as the difference between observed feed intake and expected feed intake, the latter based on the animal’s rate of gain and body back fat content [9, 10]. Animals with lower RFI are more efficient and animals with high RFI are less efficient.

Thus, measuring feed efficiency poses significant challenges that are often ignored in both research and commercial settings. Failure to appreciate basic factors affecting the measurement of feed efficiency can lead to incorrect conclusions in the interpretation of data.

### Expressing feed efficiency

Feed efficiency has traditionally been expressed on the basis of a ratio of feed consumed and growth achieved. More recently, other expressions of feed efficiency have been adopted. The selection of the correct term is generally dictated by the knowledge being sought and the manner in which the information will be used to make decisions. It should also be expressed on the basis of data in which there is the greatest confidence. The problem of feed wastage, previously discussed, is an obvious example.

A relatively simple, but increasingly common, modification is the calculation of feed conversion on the basis of carcass gain as opposed to total bodyweight gain [6, 11, 12]. This correction has evolved with the increasing use of higher fiber diets which are associated with changes in dressing percentage concurrent with pigs being sold – and paid for – on a carcass weight basis. Consequently, differences in dressing percentage can have a substantial impact on net income. Pigs adapt to the feeding of higher fiber diets by increasing viscera weight and volume [13]; this not only alters carcass dressing percentage but it also increases energy and amino acid requirements, which further impairs feed efficiency [14, 15]. One of the challenges of expressing feed efficiency on the basis of carcass gain is access to data on dressing percentage at both the start and the end of the study. The latter is easily measured directly, but the former is often estimated, incorrectly, as the same dressing percentage measured at harvest. Generally speaking, dressing percentage increases as the pig grows, due to the fact that the total body of the pig grows at a higher proportion than the viscera [16, 17].

Feed efficiency can also be expressed on the basis of energy consumed rather than feed consumed [1]. This approach has the advantage of placing increased focus on the efficiency with which dietary energy is used by the pig. This, in turn, is valuable because energy is by far the greatest contributor to the cost of the diet [2]. However, it also has its weaknesses, not the least of which is the inaccuracy of estimating the concentration of energy in the diet. The first level of error occurs in quantifying dietary energy concentration, which is rarely determined biologically and all too often is a book value that may or may not reflect the true energy content of the ingredient (s) in the diet. The second level of error is the method of expressing dietary energy concentration. Much of the world continues to use digestible energy or metabolisable energy, despite the fact that both contain errors due to the variation in efficiency with which differing sources of energy – protein, fat, fiber and starch – are used for maintenance and for gain [8]. It is therefore very conceivable that differences observed in feed efficiency are simply artefacts of an inaccurate energy system rather than being due to any real change in efficiency with which the pig uses available energy.

Increasingly, there is a desire to represent feed efficiency in financial terms because fundamentally, the goal of pork production is to use feed resources most efficiently and effectively, and that means generating the most favorable net income. Thus, preferred expressions of feed efficiency in financial terms include feed cost per pig sold, feed cost per kg of live weight gain, feed cost per kg of carcass weight gain and return over feed cost. None of these calculations addresses the impact of growth rate on financial efficiency; the solution is to expresses each of the above on a per pig place, rather than per pig basis.

### Biological basis of feed efficiency

#### Energetics and the utilization of energy for maintenance versus gain

Maintenance is a significant component of daily energy intake in the pig. It has been estimated that in a typical 70 kg pig offered feed on an ad libitum basis, about 34 % of daily energy intake is directed to maintenance [2]. Consequently, minimizing maintenance costs to maximize the proportion of daily nutrient intake directed towards growth will improve feed efficiency and therefore must be a target in pig meat production. This will include minimizing the energetic cost of unnecessary social stress, maintaining the pig in its thermo-neutral zone, and minimizing the impact of disease on the pig [1]. Most of the efforts directed at increasing efficiency in pig meat production have focused on genetic selection towards increased leanness. This effect, however, will reach a plateau when economically optimum levels of leanness in the pig carcass are achieved. Therefore, further efficiency increases may also come from a reduction in overhead costs, either by a further increase in growth rate (reduction of the time to reach harvest weight), efficient manipulation of nutrient partitioning towards growth (e.g., regulators of nutrient partitioning such as somatotropin), or by decreasing the overall maintenance requirements per unit of metabolic body weight of a growing pig [18]. However, there is no universal agreement in the literature on the influence of the production system on maintenance requirements, and consequently, the importance of reducing maintenance costs for improvements in feed efficiency. This was illustrated by Bauman et al. who concluded that selection based on genetic merit for milk production in dairy cows does not influence the maintenance requirement, and that differences in the maintenance requirement per unit of metabolic body size represent only a small component of the differences in productive efficiency among cows [19, 20].

#### Effect of feed intake on composition of gain

Growth requires a substantial quantity of nutrients to support tissue maintenance and deposition. Before estimating any nutritional requirement of a given pig genotype, it is essential to understand the process of growth, which will in turn dictate the requirement. Feed intake is important to consider because it dictates the magnitude of changes in daily growth of lean and fatty tissues, and carcass quality in meat-producing animals [21]. Protein growth responds linearly to feed intake up to a maximum point at which it plateaus, the so-called PD_max_ [22, 23]. Pigs with high feed intakes reach the plateau earlier in life, and daily nitrogen retention is therefore constant over a wide range of live weights thereafter [24]. Similar observations have been reported in male turkeys where growth rate was accelerated to a plateau early in life, and this rate did not further increase but held at a relatively constant daily gain until reaching 70 % of the mature body weight [21]. Lean growth increases linearly with feed supply but reaches a plateau at the maximum lean growth potential of the animal. However, improved genotypes or different sexes may show greater growth rates. The entire male, for example, has a much greater potential for lean tissue growth than either the female or the castrated male [25]. These differences can only be seen at greater feed intakes. In poultry and in ruminants, differences in growth rates are also influenced by sex and are much more evident at high feed intakes [22].

During the growth phase when there is a linear lean accretion response to energy intake, it is believed that the animal prefers to target lean while maintaining a minimal level of fat gain [21]. Certainly, this is how most models partition dietary energy; after maintenance needs are accounted for, and the energy required for lean accretion is determined, the remaining energy is assigned to lipid accretion. In this way, lipid accretion rates can be estimated for any genotype in which the protein deposition curve is known and daily energy intake is also known [26]. However, when energy intake is severely restricted, a minimum lipid:protein accretion ratio is observed [27]; this appears to be genetically coded. As energy intake increases, both protein and fat deposition will increase. The rate of protein accretion per unit of energy intake is typically linear until PD_max_ is achieved. This is often the point of maximum production efficiency, because growth rate is high and because if energy intake further increases, only lipid is deposited onto the carcass [17, 26].

#### Residual feed intake

Residual feed intake (RFI) is a measure of production efficiency defined as the difference between the observed and expected feed intake of an individual based on growth and backfat [28]. Pigs divergently selected for RFI consistently demonstrate differences in carcass composition and in feed intake. Low RFI pigs had less carcass fat, consume less feed and exhibit similar or slightly slower rates of gain compared with high RFI pigs [10, 28, 29]. In the Iowa State University herd, seven generations of selection for low RFI resulted in a reduction in ADFI of 0.6 kg/d with only a modest decline in growth rate compared to the high RFI line. This corresponds to an increase in feed efficiency of 35 % [28]. Harris et al. reported that selection for low RFI alters nutrient utilization and energy digestibility, as well as improving nitrogen and phosphorus balance [30].

The flux through pathways of protein degradation, which include calpain and the ubiquitin-proteasomal system within muscle, are decreased in the low RFI line [31]. Additionally, genetic selection for low RFI may result in a decrease in oxidative stress due to a reduction in both electron leakage and production of reactive oxygen species from mitochondria in muscle and liver tissues [32]. Therefore, part of the improvement in feed efficiency in low RFI pigs could be explained by the lower rate of protein degradation and by reduced oxidative stress.

#### Carcass composition and meat quality

Concern has been expressed that pigs highly selected for improved feed efficiency may produce pork which is of inferior quality. Compared to a random control, carcasses of animals selected for low RFI had [29, 33, 34] or tended to have less back fat [28, 35]. French data indicated that selection for low RFI lowered post mortem pH and resulted in slightly poorer meat quality [36]. The French researchers also reported that water holding capacity was reduced [33] and sensory quality was impaired [34] in low RFI pigs. These results differ somewhat from that reported by Iowa State University researchers [35]. Smith et al. found that pork from low RFI pigs did not differ from controls with respect to drip loss or purge loss and expressed minimal color changes. However, the authors also reported a correlation between selection for RFI and decreased sensory traits related to reduced intramuscular lipid and a decrease in post mortem proteolysis of myofibrillar proteins such as desmin.

#### Susceptibility to immunological stress

It is well known that pigs exposed to pathogens respond with reduced feed intake and consequently growth rate [37, 38]. Exposure of an animal to pathogens triggers a shift in its metabolic priorities to mount an appropriate immune response. Under pathogenic challenge, pigs require nutrients for functions that enable them to defend; some of these functions include: 1) innate immune response, 2) replenishment of damaged or lost tissue (plasma, sloughed cells etc.) and 3) acquired immune response [39]. Under scarce resources due to lower feed intake, the pig needs to allocate resources to fight the pathogens and operate the normal functions of a healthy pig (e.g., maintenance and growth). One important fact to consider is that protein becomes the first limiting resource in immune challenged pigs because many components of the immune response are rich in proteins [39]. It is also generally accepted that energy requirements are lower for growing animals during a health challenge [40], but there is also evidence of energy becoming limiting in pathogen-challenged pigs because of an increase in heat production (fever) and activation of the immune response [39]. The functions of immunity may increase the maintenance requirement, and are prioritized over functions of growth in terms of nutrient allocation [40].

It has been theorized by some that selection for improved efficiency might make pigs more susceptible to disease. Rakhshandeh et al. evaluated the impact of a repeated LPS (lipopolysaccharide) challenge on both high and low RFI lines [41]. They observed no differences in apparent ileal digestibility of nutrients (AID), but did observe an increase in apparent total tract digestibility (ATTD) under this modelled immune challenge. They did not see any impact on intestinal nutrient transport or barrier function. Divergent selection for low RFI increased apparent total tract digestibility of nutrients, but it had no effect on apparent ileal digestibility of nutrients. However, immune system stimulation affected both AID and ATTD of dietary nutrients and may be a major source of variation in feed efficiency among individuals. Altogether, genetic selection for LRFI appears to reduce the total tract digestive capacity of growing pigs during immune system stimulation. However, additional data are required to confirm these findings.

### Improving feed efficiency

**Fig. 1:**
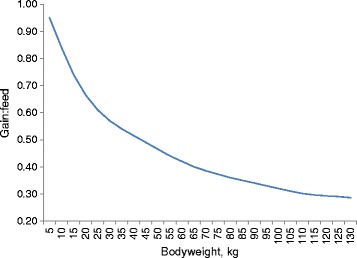
Relationship between body weight and feed conversion

Feed efficiency is a function of body weight [42], so as the pig grows toward market weight, it becomes less efficient at converting feed into body weight gain (Fig. 1). Nonetheless, at a given weight, feed conversion can be affected by numerous internal and external influences, as previously described. Understanding and controlling these elements provides the scientific foundation for achieving improvements in feed efficiency. Internal factors of particular interest include maintenance processes, body composition and health status. External factors include the nutrient and ingredient composition of the feed, the manner in which the feed has been processed, the thermal environment in which the pig lives, access to feed and water and the use of various feed additives.

#### Internal factors

About one-third of the variation in feed conversion among animals is related to processes unrelated to the rate and composition of growth [43]. Consequently, efforts directed at improving feed conversion must consider these maintenance processes. Some, such as basal metabolic rate and body protein turnover rate, are under at least partial genetic control [43]. Others are related to activity, which van Milgen et al. [44] reported to be proportional to protein mass in the body, and thermoregulation which changes in a curvilinear manner according to the deviation from the pig’s thermal comfort zone [45].

Immune function is often considered a component of maintenance requirements, although classical definitions exclude it. Nonetheless, host-pathogen interactions place significant energy demands on the animal as a consequence of a multi-faceted response to infection [46]. Minimizing demands placed on the pig’s immune system is an effective way to improve feed efficiency [47].

Body composition is fundamentally a function of sex and genotype, along with prior nutritional regime and health status. It is well documented that entire males are more efficient than females, which in turn are more efficient than castrated males [26]. However, at the present time, most producers have little control over the gender of the animals they are raising. Sex selection technology applied to sperm was first demonstrated in 1989 [48], but in swine faces considerable technical challenges before it can be applied widely [49]. Selection of pigs with no or reduced androstenone, along with the implementation of dietary regimes that minimize skatole production, the two essential contributors to boar taint, is another approach being pursued [50]. Finally, immunological castration offers another tool to permit the feeding of more efficient intact males rather than castrates while addressing concerns about pork quality and behavior [51]. At the present time, there appears to be limited market penetration for this technology, but it has achieved success in selected markets. Therefore, at the present time commercial pork production will continue to be based on traditional castrated males and females, although this is likely to change in the future – with attendant improvements in feed efficiency.

#### Factors external to the pig

It is well known that increasing the concentration of energy in a diet will almost always result in improved feed efficiency Table 1 [52, 53]. If no improvement is observed, it means that a nutrient deficiency is impairing the pig’s ability to respond to the energy, or that ingredient energy values are incorrect.

**Table 1:** Impact of increasing dietary energy concentration on daily energy intake and growth rate

Diet ME, Mcal/kg	2.95	3.05	3.15	3.25	3.35
Initial wt., kg	31.2	31.1	31.5	31.2	31.1
Final wt., kg	115.1	115.3	115.1	115.0	115.5
Daily gain, kg	1.00	1.01	1.03	1.03	1.03
Daily feed, kg^1^	2.80	2.66	2.64	2.61	2.47
Feed conversion^1^	2.78	2.63	2.56	2.56	2.38
ME intake, Mcal/d	8.21	8.20	8.38	8.45	8.38

^1^Effect of diet ME concentration significant, *P* < 0.05; Source: [52]

While increasing dietary energy is virtually guaranteed to improve feed efficiency, there is a surprisingly poor correlation between energy concentration in the diet and feed efficiency when measured among farms, among experiments, or among widely differing diet conditions. Oresanya et al. reported a correlation between daily DE or NE intake and feed efficiency of only 0.12 to 0.14 [54]. The reason for this discrepancy rests in the diversity of factors that can influence feed efficiency, the inaccuracy of measuring feed efficiency, the imprecision of quantifying dietary energy concentration and simple animal variation.

Restricted feed intake is practiced in some parts of the world to achieve 2 objectives: to improve carcass quality by reducing fat content, and to improve feed efficiency. This practice improves feed efficiency through the reduction in body fat, although the benefit is offset somewhat by the slower rate of growth. The energetic cost of protein deposition in the body is about 10.03 kcal ME/kg, while the comparable value for fat is 11.65 Mcal ME/kg [2]. On this basis, fat deposition requires 16 % more energy per unit of gain. However, protein accretion is accompanied by water deposition in lean gain, in a ratio of about 4:1, so the actual advantage of lean accretion is greater than 4:1 over fat [55]. In most major pork producing regions, limit feeding is not practiced because it lowers growth rate and thus barn throughput, a key contributor to overall farm profitability.

Feed restriction may also improve feed efficiency by reducing feed wastage. This occurs because pigs are more likely to waste feed if it is in abundant supply.

There can be circumstances where restricted feed intake fails to improve feed efficiency. When restriction is so severe that growth rate is seriously reduced, the additional days required to achieve market weight also increases the number of days of maintenance required, such that the savings due to improved efficiency of gain, due to an improved ratio of gain of lipid to protein, is fully offset by the energetic cost of additional days in the barn Table 2 [27]. This example illustrates the importance of conducting research to constant final body weight in order to effectively compare feed efficiency outcomes.

**Table 2:** Least square means of the impact of decreasing energy intake through feed restriction on barrow and gilt performance from 25 to 120 kg

Item	Percent of *ad libitum*					SEM	*P*-values		
	100	93	86	79	72		Treatment	Linear	Quadratic
Initial wt., kg	24.7	24.7	24.7	24.7	24.7				
Final wt., kg	120.0	118.9	118.7	119.0	119.6	0.2	0.320	0.107	0.001
Daily gain, kg/d	1.00	0.92	0.76	0.78	0.66	0.01	0.001	0.001	0.952
Daily feed, kg/d	2.64	2.44	2.25	2.06	1.87	0.02	0.001	0.001	0.829
Feed conversion	0.40	0.40	0.40	0.41	0.38	0.01	0.046	0.169	0.033
Loin, mm	61.6	55.6	55.3	57.1	57.1	0.6	0.047	0.824	0.409
Backfat, mm	16.7	14.8	13.5	12.7	12.3	0.3	0.001	0.001	0.832
Daily DE intake, Mcal/d	8.67	8.01	7.41	6.77	6.15				
Protein accretion, g/d	145.6	135.8	140.8	131.8	112.9	6.3	0.014	0.012	0.184
Lipid accretion, g/d	294.8	246.6	213.6	183.3	144.2	9.7	0.001	0.001	0.874
Ash accretion, g/d	27.1	26.7	28.6	23.2	19.3	1.8	0.010	0.005	0.085
Lipid:protein ratio	2.05	1.89	1.57	1.43	1.30	0.12	0.001	0.001	0.746
Ash:protein ratio	0.10	0.11	0.14	0.13	0.14	0.01	0.086	0.002	0.737

Source: [27]

The management of feed processing can substantially influence feed efficiency. Numerous studies have reported near linear relationships between grain particle size and feed efficiency [56–58]. There is also a suggestion that reducing the standard deviation of particle size will also improve the digestibility of the diet, although more research is clearly required on this understudied tropic Tables 3 and 4 [59].

**Table 3:** Impact of particle size standard deviation on digestibility of experimental diets

Particle Size ave, μm	545	551	564	599	545	SEM	*P* value	
Particle size SD, μm	1.88	2.11	2.33	2.51	2.73		linear	quadratic
Gross Energy, kcal/g (DM)								
-Diet	4.43	4.43	4.42	4.45	4.42			
-Feces	4.55	4.70	4.61	4.63	4.68	0.02	0.0008	0.88
Apparent Digestibility, %								
-Energy	85.79	84.18	81.12	83.37	84.96	0.63	0.31	<0.0001
-Dry Matter	85.31	84.10	81.12	82.96	84.49	0.67	0.34	<0.0001

Source: [59]

**Table 4:** Impact of particle size standard deviation on the gross energy content of diet and of digestible and metabolizable energy content of the corn, dry matter basis

Items	Particle Size Standard Deviation					SEM	*P* value	
	1.88	2.11	2.33	2.51	2.73		linear	linear
DM, %	95.62	95.53	95.35	95.59	95.57	0.18	0.83	0.24
GE, Mcal/kg	4.43	4.43	4.42	4.45	4.42			
DE, Mcal/kg	3.80	3.73	3.59	3.71	3.76	0.03	0.23	<0.0001
ME, Mcal/kg^1^	3.74	3.68	3.53	3.66	3.70	0.03	0.23	<0.0001

^1^Calculated from DE using equation of [67]

Source: [59]

The desire to maximize feed efficiency through a more finely ground feed must be balanced against the cost of the additional processing, potential difficulties with diet flowability and possible impacts on animal health, notably gastric ulcers [56]. Ulcers become a greater issue when particle size falls below about 500 microns and when diets are pelleted.

The desire to further reduce particle size to less than 600 microns has resulted in problems with the flowability of diets, which in turn impacts manufacturing, transportation and delivery of feed. As a consequence, and coincident with unusually high feed costs since 2009, many pork producers and feed companies in North America switched from mash to pelleted diets, something adopted by European producers many years earlier due to their higher feed costs.

Pelleting provides a clear benefit in terms of feed efficiency [60], but the real advantage of pelleting is confounded by particle size. It is well understood that the benefit of pelleting is greatest when particle size is large, and declines as particle size diminishes [60]. Furthermore, the value of pelleting is maximized when fines are minimized, but achieving consistent pellet quality is challenging [61]. Stark has suggested that numerous factors influence final pellet quality, and that about 40 % is related to the diet formulation, 20 % to conditioning, 20 % to particle size, 15 % to die specification and 5 % to cooling [62].

The pig, like all mammals, is a homeotherm, meaning that it can maintain - and indeed must maintain - a constant body core temperature across a relatively wide range in ambient temperature. This is achieved by adjusting heat losses and heat production such that body temperature stay largely constant. Maintenance activities in the pig provide about 70 to 72 % of total heat production at thermoneutrality [63]. The efficiency of utilization of dietary energy depends on the substrate; fat and starch are more efficient than protein and fiber. Thus, utilization of higher fiber or higher protein diets in the warm summer months is contra-indicated since they will generate more heat during metabolism. If excess heat is produced by the body of the pig, it will typically respond by reducing feed intake in an attempt to lower heat production and thus reduce the metabolic expense of dissipating heat from the body. On the other hand, if the pig is housed in chilled conditions, diets higher in protein and fiber could be beneficial due to the thermic effect of feed. Clearly, the composition of the diet, as it relates to heat increment can contribute to improvements or reductions in feed efficiency.

The pig’s environment will have a substantial impact on performance. For example, if the temperature drops below the pigs’ lower critical temperature, feed intake will increase by 1.5 % per °C [26] and feed efficiency will become poorer [64]. The lower critical temperature of the pig is estimated to be about 23 - 24 °C at 25 kg body weight, dropping to about 15 °C at 100 kg [64].

These lower critical temperatures (LCT) assume that the pigs are healthy, the floor is dry and there are no drafts. They also assume that the barn is well insulated. If any of these situations do not exist, the LCT will be increased by perhaps 2 to 3 °C to accommodate the chilling impact of dampness, drafts, etc. Also, if pigs are not healthy – and therefore not eating to their full potential – their LCT will be much higher.

The limited data that are available suggest that feed conversion is minimally affected by elevated temperatures in the barn. As pigs become heat stressed, feed intake will decline by about 1 % (growing pigs) and 2 % (finishing pigs) for every degree above the upper critical temperature [60]. The decline in feed intake is manifested in slower growth, such that changes in feed efficiency are surprisingly small [65].

Delivery of feed to the pig may also play an important role in improving feed efficiency. Poorly designed feeders, combined with poor management of the feeders, can lead to excessive feed wastage and poorer feed efficiency, or to impaired feed intake. Feeders adjusted too tightly can severely reduce feed intake and lengthen the time pigs spend at the feeder, thus reducing its capacity [66]. Feeder design will determine if tight adjustment will improve feed efficiency, by reducing feed wastage, or simply reduce feed intake and thus animal growth. Optimum feeder adjustment will depend on many factors, but for dry feeders, 40 % coverage of the feeder pan with feed is currently recommended [66].

Inadequacy of feeder space may also result in poorer feed conversion although Weber et al. reported no impact of feeder space until the pigs reached the final phase of grow-out prior to marketing [6].

## Conclusions

Feed efficiency is a critically important topic in pork production. It is also highly complex in nature, because it is affected by much more than diet composition. The utilization of energy in the diet is a foundational driver of feed efficiency. Knowledge of this is essential to the most effective management of feed efficiency. However, many other factors are also involved, such as body composition, initial and final body weight, the level of feed intake, growth rate, the thermal environment, immunological status and finally, feed processing and delivery. Given this large number of important variables, it is clear that this simple calculation of the ratio of body weight gain to feed consumed provides information that is actually very complex. Whether it is being measured in research or in commercial practice, all of the factors that potentially influence the outcome must be understood and considered.

## Abbreviations

RFI: Residual feed intake
PD: Protein deposition
RFI: Residual feed intake
ADFI: Average daily feed intake
LRFI: Low residual feed intake
AID: Apparent ileal digestible
ATTD: Apparent total tract digestible
DE: Digestible energy
NE: Net energy
ME: Metabolizable energy
LCT: Lower critical temperature
